# Bis(triphenylphosphine)iminium bromide acetonitrile monosolvate

**DOI:** 10.1107/S1600536810046337

**Published:** 2010-11-17

**Authors:** Carsten Knapp, Rabiya Uzun

**Affiliations:** a Institut für Anorganische und Analytische Chemie, Albert-Ludwigs-Universität Freiburg, Albertstrasse 21, 79104 Freiburg i. Br., Germany

## Abstract

The title compound, C_36_H_30_NP_2_^+^·Br^−^·C_2_H_3_N, crystallized from a CH_3_CN/OEt_2_ solution as an acetonitrile solvate. The central P—N—P angle [142.88 (10)°] is significantly larger than in the corresponding chloride and iodide structures.

## Related literature

Several bis­(triphenyl­phosphine)iminium halide structures have been determined. For [(Ph_3_P)_2_N]Cl, see: Knapp *et al.* (2010 ▶); for [(Ph_3_P)_2_N]Cl·B(OH)_3_, see: Andrews *et al.* (1983 ▶); for [(Ph_3_P)_2_N]Cl·CH_3_C_6_H_5_, see: Weller *et al.* (1993 ▶); for [(Ph_3_P)_2_N]Cl·CH_2_Cl_2_, see: Carroll *et al.* (1996 ▶); for [(Ph_3_P)_2_N]Cl·CH_2_Cl_2_·H_2_O, see: de Arellano (1997 ▶); for [(Ph_3_P)_2_N]I, see: Beckett *et al.* (2010 ▶). For a discussion of the [(Ph_3_P)_2_N]^+^ cation, see: Lewis *et al.* (2000 ▶). For the synthesis, see: Martinsen & Songstad (1977 ▶).
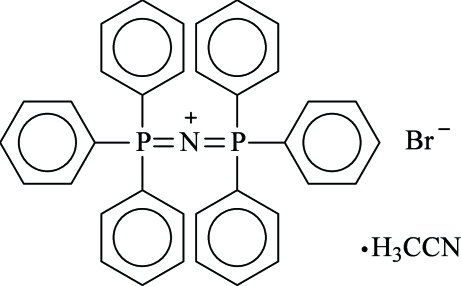


## Experimental

### 

#### Crystal data

C_36_H_30_NP_2_^+^·Br^−^·C_2_H_3_N
*M* *_r_* = 659.51
Orthorhombic, 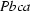

*a* = 19.7113 (6) Å
*b* = 15.9564 (5) Å
*c* = 20.3318 (6) Å
*V* = 6394.8 (3) Å^3^
*Z* = 8
Mo *K*α radiation
μ = 1.42 mm^−1^
*T* = 100 K
0.20 × 0.20 × 0.16 mm

#### Data collection

Bruker APEXII CCD area-detector diffractometer
Absorption correction: multi-scan (*SADABS*; Bruker, 2001 ▶) *T*_min_ = 0.765, *T*_max_ = 0.805
57718 measured reflections
7749 independent reflections
6140 reflections with *I* > 2σ(*I*)
*R*_int_ = 0.045

#### Refinement

*R*[*F* ^2^ > 2σ(*F* ^2^)] = 0.034
*wR*(*F* ^2^) = 0.085
*S* = 1.02
7749 reflections
391 parameters
Only H-atom displacement parameters refined
Δρ_max_ = 0.51 e Å^−3^
Δρ_min_ = −0.45 e Å^−3^

### 

Data collection: *APEX2* (Bruker, 2007 ▶); cell refinement: *SAINT* (Bruker, 2007 ▶); data reduction: *SAINT*; program(s) used to solve structure: *SHELXS97* (Sheldrick, 2008 ▶); program(s) used to refine structure: *SHELXL97* (Sheldrick, 2008 ▶); molecular graphics: *DIAMOND* (Brandenburg *et al.*, 2010 ▶); software used to prepare material for publication: *SHELXL97*.

## Supplementary Material

Crystal structure: contains datablocks I, global. DOI: 10.1107/S1600536810046337/fi2100sup1.cif

Structure factors: contains datablocks I. DOI: 10.1107/S1600536810046337/fi2100Isup2.hkl

Additional supplementary materials:  crystallographic information; 3D view; checkCIF report

## Figures and Tables

**Table:** 

P1—N1	1.5767 (16)
P1—C1	1.7942 (19)
P1—C13	1.7988 (18)
P1—C7	1.7997 (18)
P2—N1	1.5797 (15)
P2—C31	1.7963 (18)
P2—C19	1.7978 (18)
P2—C25	1.8063 (19)

**Table:** 

P1—N1—P2	142.88 (10)

**Fig. 1.**
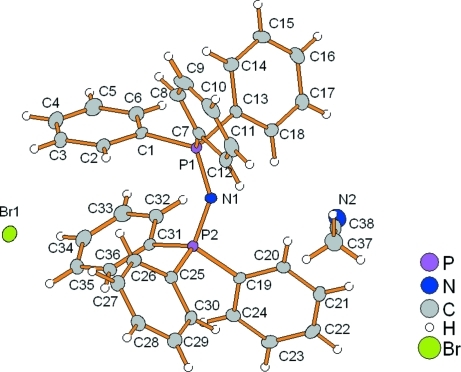
View of the asymmetric unit of [(Ph3P)2N]Br.CH3CN. Displacement ellipsoids are shown at the 50% probability level and hydrogen atoms are drawn with arbitrary radii.

## supplementary crystallographic information

### Comment

Crystal structures of [(Ph_3_P)_2_N]^+^ salts containing small counter anions are rare. Usually this cation is partnered by a bulky anion, while crystal structures containing small anions and especially halides remained unknown until recently. Only very recently the crystal structures of the halides [(Ph_3_P)_2_N]Cl (Knapp *et al.*, 2010) and [(Ph_3_P)_2_N]I (Beckett *et al.*, 2010) were determined. In contrast, crystal structures of [(Ph_3_P)_2_N]Cl containing solvate molecules are known for some time, *e.g.* [(Ph_3_P)_2_N]Cl^.^B(OH)_3_ (Andrews *et al.* (1983)), [(Ph_3_P)_2_N]Cl^.^CH_3_C_6_H_5_, (Weller *et al.* (1993)), [(Ph_3_P)_2_N]Cl^.^CH_2_Cl_2_ (Carroll *et al.* (1996)), [(Ph_3_P)_2_N]Cl^.^CH_2_Cl_2_^.^H_2_O (de Arellano (1997)).

[(Ph_3_P)_2_N]Br has been synthesized according to a published procedure (Martinsen *et al.*, 1977) and single crystals suitable for X-ray diffraction were obtained by layering a CH_3_CN solution with diethyl ether. In contrast to the the crystal structures of [(Ph_3_P)_2_N]Cl (Knapp *et al.*, 2010) and [(Ph_3_P)_2_N]I (Beckett *et al.*, 2010) the title compound crystallized with an aceteontrile solvate molecule.

The central P—N—P angle [142.89 (11)°] is significantly larger than in the corresponding chloride and iodide structures but still falls into the common range for PNP angles in these cations (Lewis *et al.*, 2000). The P-N (1.5775 (17) and 1.5790 (16) Å) and P-C distances (179.4 (2)–180.6 (2) Å) are in the expected range.

### Experimental

[(Ph_3_P)_2_N]Br has been synthesized from [(Ph_3_P)_2_N]Cl and KBr in water according to a literature method (Martinsen *et al.*, 1977). Single crystals suitable for X-ray diffraction were obtained by layering a CH_3_CN solution with diethyl ether.

### Refinement

The hydrogen atoms were positioned geometrically and refined using a riding model. The same *U_iso_* value was used for all H atoms, which refined to 0.0237 (12) Å^2^.

### Crystal data

**Table:**

C_36_H_30_NP_2_^+^·Br^−^·C_2_H_3_N	*F*(000) = 2720
*M**_r_* = 659.51	*D*_x_ = 1.370 Mg m^−^^3^
Orthorhombic, *P**b**c**a*	Mo *K*α radiation, λ = 0.71073 Å
Hall symbol: -P 2ac 2ab	Cell parameters from 9913 reflections
*a* = 19.7113 (6) Å	θ = 2.4–27.3°
*b* = 15.9564 (5) Å	µ = 1.42 mm^−^^1^
*c* = 20.3318 (6) Å	*T* = 100 K
*V* = 6394.8 (3) Å^3^	Block, colourless
*Z* = 8	0.20 × 0.20 × 0.16 mm

### Data collection

**Table:**

Bruker APEXII CCD area-detector diffractometer	7749 independent reflections
Radiation source: microfocus sealed tube	6140 reflections with *I* > 2σ(*I*)
multilayer mirro optics	*R*_int_ = 0.045
φ and ω scans	θ_max_ = 28.1°, θ_min_ = 2.1°
Absorption correction: multi-scan (*SADABS*; Bruker, 2001)	*h* = −26→21
*T*_min_ = 0.765, *T*_max_ = 0.805	*k* = −21→20
57718 measured reflections	*l* = −25→26

### Refinement

**Table:**

Refinement on *F*^2^	Primary atom site location: structure-invariant direct methods
Least-squares matrix: full	Secondary atom site location: difference Fourier map
*R*[*F*^2^ > 2σ(*F*^2^)] = 0.034	Hydrogen site location: inferred from neighbouring sites
*wR*(*F*^2^) = 0.085	Only H-atom displacement parameters refined
*S* = 1.02	*w* = 1/[σ^2^(*F*_o_^2^) + (0.0378*P*)^2^ + 4.5281*P*] where *P* = (*F*_o_^2^ + 2*F*_c_^2^)/3
7749 reflections	(Δ/σ)_max_ < 0.001
391 parameters	Δρ_max_ = 0.51 e Å^−^^3^
0 restraints	Δρ_min_ = −0.45 e Å^−^^3^

### Special details

**Table:**

Geometry. All e.s.d.'s (except the e.s.d. in the dihedral angle between two l.s. planes) are estimated using the full covariance matrix. The cell e.s.d.'s are taken into account individually in the estimation of e.s.d.'s in distances, angles and torsion angles; correlations between e.s.d.'s in cell parameters are only used when they are defined by crystal symmetry. An approximate (isotropic) treatment of cell e.s.d.'s is used for estimating e.s.d.'s involving l.s. planes.
Refinement. Refinement of *F*^2^ against ALL reflections. The weighted *R*-factor *wR* and goodness of fit *S* are based on *F*^2^, conventional *R*-factors *R* are based on *F*, with *F* set to zero for negative *F*^2^. The threshold expression of *F*^2^ > σ(*F*^2^) is used only for calculating *R*-factors(gt) *etc*. and is not relevant to the choice of reflections for refinement. *R*-factors based on *F*^2^ are statistically about twice as large as those based on *F*, and *R*- factors based on ALL data will be even larger.

### Fractional atomic coordinates and isotropic or equivalent isotropic displacement parameters (Å^2^)

**Table:**

	*x*	*y*	*z*	*U*_iso_*/*U*_eq_	
P1	0.25018 (2)	0.06051 (3)	0.17193 (2)	0.01189 (10)	
N1	0.18271 (8)	0.01276 (9)	0.15375 (7)	0.0143 (3)	
P2	0.12559 (2)	0.00609 (3)	0.09951 (2)	0.01196 (10)	
C1	0.28765 (10)	0.12141 (11)	0.10725 (9)	0.0148 (4)	
C2	0.25987 (10)	0.19901 (12)	0.09087 (9)	0.0190 (4)	
H2	0.2253	0.2228	0.1176	0.0236 (11)*	
C3	0.28261 (11)	0.24142 (13)	0.03571 (11)	0.0253 (5)	
H3	0.2628	0.2935	0.0238	0.0236 (11)*	
C4	0.33434 (12)	0.20749 (14)	−0.00204 (10)	0.0285 (5)	
H4	0.3503	0.2368	−0.0397	0.0236 (11)*	
C5	0.36285 (12)	0.13154 (14)	0.01468 (10)	0.0262 (5)	
H5	0.3990	0.1095	−0.0110	0.0236 (11)*	
C6	0.33941 (10)	0.08711 (12)	0.06833 (9)	0.0192 (4)	
H6	0.3581	0.0339	0.0788	0.0236 (11)*	
C7	0.23573 (10)	0.12855 (11)	0.24112 (9)	0.0144 (4)	
C8	0.28104 (10)	0.19243 (12)	0.25734 (10)	0.0190 (4)	
H8	0.3185	0.2041	0.2294	0.0236 (11)*	
C9	0.27146 (11)	0.23902 (13)	0.31445 (10)	0.0251 (5)	
H9	0.3020	0.2830	0.3253	0.0236 (11)*	
C10	0.21734 (12)	0.22111 (13)	0.35532 (10)	0.0262 (5)	
H10	0.2117	0.2517	0.3950	0.0236 (11)*	
C11	0.17135 (12)	0.15921 (13)	0.33905 (10)	0.0244 (5)	
H11	0.1336	0.1486	0.3669	0.0236 (11)*	
C12	0.18020 (10)	0.11242 (12)	0.28210 (9)	0.0184 (4)	
H12	0.1487	0.0696	0.2710	0.0236 (11)*	
C13	0.31240 (9)	−0.01526 (11)	0.19747 (8)	0.0131 (4)	
C14	0.37648 (10)	0.01108 (12)	0.21822 (9)	0.0181 (4)	
H14	0.3871	0.0692	0.2192	0.0236 (11)*	
C15	0.42468 (10)	−0.04721 (13)	0.23733 (9)	0.0204 (4)	
H15	0.4683	−0.0291	0.2513	0.0236 (11)*	
C16	0.40922 (11)	−0.13207 (12)	0.23604 (9)	0.0201 (4)	
H16	0.4424	−0.1721	0.2489	0.0236 (11)*	
C17	0.34575 (11)	−0.15843 (12)	0.21612 (10)	0.0203 (4)	
H17	0.3352	−0.2165	0.2159	0.0236 (11)*	
C18	0.29719 (10)	−0.10029 (12)	0.19634 (9)	0.0170 (4)	
H18	0.2538	−0.1187	0.1821	0.0236 (11)*	
C19	0.08890 (9)	−0.09672 (11)	0.10613 (9)	0.0127 (4)	
C20	0.09094 (10)	−0.13784 (11)	0.16677 (9)	0.0159 (4)	
H20	0.1147	−0.1138	0.2028	0.0236 (11)*	
C21	0.05793 (10)	−0.21417 (12)	0.17393 (10)	0.0193 (4)	
H21	0.0594	−0.2427	0.2149	0.0236 (11)*	
C22	0.02288 (10)	−0.24900 (12)	0.12149 (10)	0.0212 (4)	
H22	−0.0002	−0.3008	0.1270	0.0236 (11)*	
C23	0.02120 (10)	−0.20880 (12)	0.06107 (10)	0.0204 (4)	
H23	−0.0025	−0.2333	0.0252	0.0236 (11)*	
C24	0.05428 (10)	−0.13253 (12)	0.05318 (9)	0.0171 (4)	
H24	0.0533	−0.1048	0.0118	0.0236 (11)*	
C25	0.05703 (9)	0.07942 (12)	0.11228 (9)	0.0146 (4)	
C26	0.07040 (10)	0.16532 (12)	0.11015 (10)	0.0189 (4)	
H26	0.1149	0.1845	0.1004	0.0236 (11)*	
C27	0.01909 (10)	0.22260 (12)	0.12217 (9)	0.0200 (4)	
H27	0.0285	0.2810	0.1207	0.0236 (11)*	
C28	−0.04602 (10)	0.19508 (12)	0.13630 (9)	0.0186 (4)	
H28	−0.0809	0.2345	0.1454	0.0236 (11)*	
C29	−0.06021 (10)	0.10991 (13)	0.13709 (9)	0.0201 (4)	
H29	−0.1050	0.0911	0.1458	0.0236 (11)*	
C30	−0.00886 (10)	0.05211 (12)	0.12510 (9)	0.0168 (4)	
H30	−0.0187	−0.0062	0.1257	0.0236 (11)*	
C31	0.15659 (9)	0.02082 (11)	0.01724 (8)	0.0135 (4)	
C32	0.21624 (10)	−0.02015 (12)	−0.00108 (9)	0.0185 (4)	
H32	0.2388	−0.0557	0.0294	0.0236 (11)*	
C33	0.24261 (11)	−0.00892 (13)	−0.06364 (9)	0.0230 (4)	
H33	0.2834	−0.0365	−0.0760	0.0236 (11)*	
C34	0.20953 (11)	0.04257 (13)	−0.10835 (10)	0.0243 (5)	
H34	0.2276	0.0500	−0.1513	0.0236 (11)*	
C35	0.15028 (11)	0.08313 (13)	−0.09059 (9)	0.0227 (4)	
H35	0.1276	0.1181	−0.1214	0.0236 (11)*	
C36	0.12382 (10)	0.07294 (12)	−0.02784 (9)	0.0178 (4)	
H36	0.0834	0.1014	−0.0156	0.0236 (11)*	
Br1	0.115207 (11)	0.361552 (13)	0.016157 (10)	0.02274 (7)	
C37	0.01999 (12)	0.99644 (14)	0.40311 (11)	0.0289 (5)	
H37A	−0.0050	1.0350	0.3744	0.045 (4)*	
H37B	0.0460	1.0287	0.4355	0.045 (4)*	
H37C	−0.0121	0.9597	0.4260	0.045 (4)*	
C38	0.06723 (12)	0.94476 (13)	0.36271 (9)	0.0217 (4)	
N2	0.10065 (10)	0.90568 (13)	0.33284 (9)	0.0323 (4)	

### Atomic displacement parameters (Å^2^)

**Table:**

	*U*^11^	*U*^22^	*U*^33^	*U*^12^	*U*^13^	*U*^23^
P1	0.0114 (2)	0.0112 (2)	0.0131 (2)	−0.00099 (19)	−0.00009 (17)	0.00014 (16)
N1	0.0126 (8)	0.0153 (8)	0.0150 (8)	−0.0022 (6)	−0.0023 (6)	0.0019 (6)
P2	0.0114 (2)	0.0114 (2)	0.0131 (2)	−0.00064 (18)	−0.00081 (17)	0.00066 (16)
C1	0.0138 (10)	0.0147 (9)	0.0161 (9)	−0.0044 (8)	0.0001 (7)	−0.0001 (7)
C2	0.0166 (10)	0.0158 (10)	0.0245 (10)	−0.0016 (8)	−0.0001 (8)	0.0019 (7)
C3	0.0261 (12)	0.0195 (10)	0.0304 (11)	−0.0051 (9)	−0.0050 (9)	0.0084 (8)
C4	0.0317 (13)	0.0328 (12)	0.0209 (10)	−0.0121 (11)	−0.0003 (9)	0.0069 (9)
C5	0.0249 (12)	0.0338 (13)	0.0199 (11)	−0.0031 (10)	0.0081 (8)	−0.0017 (8)
C6	0.0201 (11)	0.0181 (10)	0.0193 (10)	−0.0005 (8)	0.0007 (8)	−0.0029 (7)
C7	0.0151 (10)	0.0133 (9)	0.0148 (9)	0.0036 (7)	−0.0026 (7)	−0.0004 (7)
C8	0.0145 (10)	0.0167 (10)	0.0257 (10)	0.0027 (8)	−0.0035 (7)	−0.0034 (8)
C9	0.0236 (11)	0.0199 (11)	0.0318 (12)	0.0072 (9)	−0.0122 (9)	−0.0096 (8)
C10	0.0364 (13)	0.0244 (11)	0.0179 (10)	0.0156 (10)	−0.0057 (9)	−0.0058 (8)
C11	0.0295 (12)	0.0260 (11)	0.0177 (10)	0.0102 (10)	0.0045 (8)	0.0033 (8)
C12	0.0198 (11)	0.0170 (9)	0.0184 (10)	0.0008 (8)	0.0029 (7)	0.0027 (7)
C13	0.0132 (9)	0.0143 (9)	0.0117 (8)	0.0018 (7)	0.0006 (6)	0.0000 (7)
C14	0.0171 (10)	0.0165 (10)	0.0208 (10)	0.0000 (8)	−0.0003 (7)	−0.0033 (7)
C15	0.0140 (10)	0.0272 (11)	0.0198 (10)	0.0009 (9)	−0.0026 (7)	−0.0036 (8)
C16	0.0218 (11)	0.0209 (10)	0.0176 (10)	0.0078 (9)	−0.0001 (8)	0.0007 (7)
C17	0.0234 (11)	0.0141 (9)	0.0233 (10)	0.0024 (8)	0.0005 (8)	0.0015 (7)
C18	0.0149 (10)	0.0168 (9)	0.0192 (9)	−0.0005 (8)	0.0005 (7)	0.0004 (7)
C19	0.0094 (9)	0.0107 (9)	0.0181 (9)	0.0005 (7)	0.0010 (7)	−0.0006 (7)
C20	0.0119 (9)	0.0172 (9)	0.0185 (9)	0.0011 (8)	−0.0006 (7)	0.0000 (7)
C21	0.0157 (10)	0.0165 (10)	0.0257 (10)	0.0027 (8)	0.0033 (8)	0.0060 (8)
C22	0.0152 (10)	0.0132 (9)	0.0353 (11)	−0.0010 (8)	0.0036 (8)	0.0000 (8)
C23	0.0171 (10)	0.0162 (10)	0.0279 (11)	−0.0007 (8)	−0.0036 (8)	−0.0048 (8)
C24	0.0155 (10)	0.0168 (9)	0.0189 (10)	0.0023 (8)	−0.0019 (7)	−0.0015 (7)
C25	0.0147 (9)	0.0149 (9)	0.0142 (9)	−0.0001 (8)	−0.0011 (7)	−0.0003 (7)
C26	0.0149 (10)	0.0165 (9)	0.0254 (10)	−0.0004 (8)	0.0000 (8)	0.0005 (7)
C27	0.0229 (11)	0.0128 (9)	0.0242 (10)	0.0008 (8)	−0.0009 (8)	−0.0003 (7)
C28	0.0200 (11)	0.0194 (10)	0.0165 (9)	0.0050 (8)	0.0012 (7)	0.0003 (7)
C29	0.0162 (10)	0.0231 (10)	0.0210 (10)	0.0012 (9)	0.0038 (8)	0.0020 (8)
C30	0.0171 (10)	0.0153 (9)	0.0182 (9)	−0.0011 (8)	0.0014 (7)	0.0018 (7)
C31	0.0132 (9)	0.0130 (9)	0.0141 (9)	−0.0031 (7)	−0.0002 (7)	−0.0006 (7)
C32	0.0202 (11)	0.0155 (10)	0.0197 (9)	0.0008 (8)	−0.0018 (7)	−0.0009 (7)
C33	0.0232 (11)	0.0251 (11)	0.0205 (10)	−0.0002 (9)	0.0051 (8)	−0.0075 (8)
C34	0.0311 (12)	0.0271 (11)	0.0147 (10)	−0.0100 (10)	0.0034 (8)	−0.0015 (8)
C35	0.0278 (12)	0.0241 (11)	0.0163 (10)	−0.0072 (9)	−0.0061 (8)	0.0047 (8)
C36	0.0155 (10)	0.0195 (10)	0.0183 (9)	−0.0008 (8)	−0.0031 (7)	0.0028 (7)
Br1	0.02371 (12)	0.02182 (11)	0.02270 (11)	−0.00315 (9)	0.00138 (8)	0.00234 (8)
C37	0.0328 (13)	0.0269 (12)	0.0270 (11)	0.0017 (10)	−0.0026 (9)	−0.0020 (9)
C38	0.0340 (13)	0.0179 (10)	0.0133 (9)	−0.0094 (9)	−0.0076 (8)	0.0045 (8)
N2	0.0332 (12)	0.0361 (11)	0.0276 (10)	−0.0023 (10)	−0.0035 (8)	0.0065 (9)

### Geometric parameters (Å, °)

**Table:**

P1—N1	1.5767 (16)	C18—H18	0.9500
P1—C1	1.7942 (19)	C19—C24	1.397 (3)
P1—C13	1.7988 (18)	C19—C20	1.397 (3)
P1—C7	1.7997 (18)	C20—C21	1.389 (3)
N1—P2	1.5797 (15)	C20—H20	0.9500
P2—C31	1.7963 (18)	C21—C22	1.387 (3)
P2—C19	1.7978 (18)	C21—H21	0.9500
P2—C25	1.8063 (19)	C22—C23	1.386 (3)
C1—C2	1.394 (3)	C22—H22	0.9500
C1—C6	1.402 (3)	C23—C24	1.390 (3)
C2—C3	1.384 (3)	C23—H23	0.9500
C2—H2	0.9500	C24—H24	0.9500
C3—C4	1.386 (3)	C25—C30	1.395 (3)
C3—H3	0.9500	C25—C26	1.396 (3)
C4—C5	1.378 (3)	C26—C27	1.385 (3)
C4—H4	0.9500	C26—H26	0.9500
C5—C6	1.381 (3)	C27—C28	1.387 (3)
C5—H5	0.9500	C27—H27	0.9500
C6—H6	0.9500	C28—C29	1.388 (3)
C7—C8	1.395 (3)	C28—H28	0.9500
C7—C12	1.399 (3)	C29—C30	1.391 (3)
C8—C9	1.392 (3)	C29—H29	0.9500
C8—H8	0.9500	C30—H30	0.9500
C9—C10	1.382 (3)	C31—C32	1.396 (3)
C9—H9	0.9500	C31—C36	1.396 (3)
C10—C11	1.381 (3)	C32—C33	1.386 (3)
C10—H10	0.9500	C32—H32	0.9500
C11—C12	1.389 (3)	C33—C34	1.388 (3)
C11—H11	0.9500	C33—H33	0.9500
C12—H12	0.9500	C34—C35	1.383 (3)
C13—C18	1.390 (3)	C34—H34	0.9500
C13—C14	1.397 (3)	C35—C36	1.388 (3)
C14—C15	1.385 (3)	C35—H35	0.9500
C14—H14	0.9500	C36—H36	0.9500
C15—C16	1.388 (3)	C37—C38	1.491 (3)
C15—H15	0.9500	C37—H37A	0.9800
C16—C17	1.381 (3)	C37—H37B	0.9800
C16—H16	0.9500	C37—H37C	0.9800
C17—C18	1.392 (3)	C38—N2	1.092 (3)
C17—H17	0.9500		
			
N1—P1—C1	115.92 (8)	C13—C18—C17	119.83 (18)
N1—P1—C13	108.54 (8)	C13—C18—H18	120.1
C1—P1—C13	107.16 (9)	C17—C18—H18	120.1
N1—P1—C7	109.95 (9)	C24—C19—C20	120.13 (17)
C1—P1—C7	108.14 (9)	C24—C19—P2	120.81 (14)
C13—P1—C7	106.72 (8)	C20—C19—P2	118.88 (14)
P1—N1—P2	142.88 (10)	C21—C20—C19	119.40 (18)
N1—P2—C31	113.51 (8)	C21—C20—H20	120.3
N1—P2—C19	107.22 (8)	C19—C20—H20	120.3
C31—P2—C19	109.03 (8)	C22—C21—C20	120.29 (18)
N1—P2—C25	112.90 (8)	C22—C21—H21	119.9
C31—P2—C25	107.67 (8)	C20—C21—H21	119.9
C19—P2—C25	106.22 (9)	C23—C22—C21	120.52 (19)
C2—C1—C6	119.84 (17)	C23—C22—H22	119.7
C2—C1—P1	119.63 (14)	C21—C22—H22	119.7
C6—C1—P1	120.11 (14)	C22—C23—C24	119.75 (18)
C3—C2—C1	120.03 (19)	C22—C23—H23	120.1
C3—C2—H2	120.0	C24—C23—H23	120.1
C1—C2—H2	120.0	C23—C24—C19	119.90 (18)
C2—C3—C4	119.7 (2)	C23—C24—H24	120.1
C2—C3—H3	120.1	C19—C24—H24	120.1
C4—C3—H3	120.1	C30—C25—C26	119.23 (18)
C5—C4—C3	120.44 (19)	C30—C25—P2	121.42 (14)
C5—C4—H4	119.8	C26—C25—P2	119.35 (14)
C3—C4—H4	119.8	C27—C26—C25	120.30 (18)
C4—C5—C6	120.7 (2)	C27—C26—H26	119.9
C4—C5—H5	119.7	C25—C26—H26	119.9
C6—C5—H5	119.7	C26—C27—C28	120.23 (18)
C5—C6—C1	119.27 (19)	C26—C27—H27	119.9
C5—C6—H6	120.4	C28—C27—H27	119.9
C1—C6—H6	120.4	C27—C28—C29	119.95 (18)
C8—C7—C12	119.63 (17)	C27—C28—H28	120.0
C8—C7—P1	121.62 (15)	C29—C28—H28	120.0
C12—C7—P1	118.57 (14)	C28—C29—C30	120.05 (19)
C9—C8—C7	120.05 (19)	C28—C29—H29	120.0
C9—C8—H8	120.0	C30—C29—H29	120.0
C7—C8—H8	120.0	C29—C30—C25	120.22 (18)
C10—C9—C8	119.7 (2)	C29—C30—H30	119.9
C10—C9—H9	120.1	C25—C30—H30	119.9
C8—C9—H9	120.1	C32—C31—C36	119.58 (17)
C11—C10—C9	120.70 (19)	C32—C31—P2	118.27 (14)
C11—C10—H10	119.6	C36—C31—P2	122.13 (15)
C9—C10—H10	119.6	C33—C32—C31	120.02 (18)
C10—C11—C12	120.1 (2)	C33—C32—H32	120.0
C10—C11—H11	119.9	C31—C32—H32	120.0
C12—C11—H11	119.9	C32—C33—C34	120.1 (2)
C11—C12—C7	119.72 (19)	C32—C33—H33	120.0
C11—C12—H12	120.1	C34—C33—H33	120.0
C7—C12—H12	120.1	C35—C34—C33	120.18 (18)
C18—C13—C14	119.61 (17)	C35—C34—H34	119.9
C18—C13—P1	120.29 (14)	C33—C34—H34	119.9
C14—C13—P1	120.10 (14)	C34—C35—C36	120.16 (19)
C15—C14—C13	120.20 (18)	C34—C35—H35	119.9
C15—C14—H14	119.9	C36—C35—H35	119.9
C13—C14—H14	119.9	C35—C36—C31	119.96 (19)
C14—C15—C16	119.94 (19)	C35—C36—H36	120.0
C14—C15—H15	120.0	C31—C36—H36	120.0
C16—C15—H15	120.0	C38—C37—H37A	109.5
C17—C16—C15	120.11 (19)	C38—C37—H37B	109.5
C17—C16—H16	119.9	H37A—C37—H37B	109.5
C15—C16—H16	119.9	C38—C37—H37C	109.5
C16—C17—C18	120.31 (18)	H37A—C37—H37C	109.5
C16—C17—H17	119.8	H37B—C37—H37C	109.5
C18—C17—H17	119.8	N2—C38—C37	178.4 (2)
			
C1—P1—N1—P2	−12.2 (2)	C14—C13—C18—C17	0.3 (3)
C13—P1—N1—P2	−132.79 (17)	P1—C13—C18—C17	179.90 (14)
C7—P1—N1—P2	110.82 (18)	C16—C17—C18—C13	−0.8 (3)
P1—N1—P2—C31	35.6 (2)	N1—P2—C19—C24	−158.31 (15)
P1—N1—P2—C19	156.03 (17)	C31—P2—C19—C24	−35.04 (18)
P1—N1—P2—C25	−87.33 (19)	C25—P2—C19—C24	80.74 (16)
N1—P1—C1—C2	77.13 (17)	N1—P2—C19—C20	26.59 (17)
C13—P1—C1—C2	−161.53 (15)	C31—P2—C19—C20	149.86 (15)
C7—P1—C1—C2	−46.83 (17)	C25—P2—C19—C20	−94.37 (16)
N1—P1—C1—C6	−95.43 (17)	C24—C19—C20—C21	−0.4 (3)
C13—P1—C1—C6	25.91 (18)	P2—C19—C20—C21	174.72 (14)
C7—P1—C1—C6	140.62 (15)	C19—C20—C21—C22	−0.4 (3)
C6—C1—C2—C3	1.0 (3)	C20—C21—C22—C23	1.0 (3)
P1—C1—C2—C3	−171.53 (16)	C21—C22—C23—C24	−0.7 (3)
C1—C2—C3—C4	−1.8 (3)	C22—C23—C24—C19	−0.1 (3)
C2—C3—C4—C5	0.6 (3)	C20—C19—C24—C23	0.7 (3)
C3—C4—C5—C6	1.5 (3)	P2—C19—C24—C23	−174.36 (15)
C4—C5—C6—C1	−2.2 (3)	N1—P2—C25—C30	−116.56 (16)
C2—C1—C6—C5	0.9 (3)	C31—P2—C25—C30	117.35 (16)
P1—C1—C6—C5	173.48 (16)	C19—P2—C25—C30	0.67 (17)
N1—P1—C7—C8	−164.10 (15)	N1—P2—C25—C26	62.58 (17)
C1—P1—C7—C8	−36.63 (18)	C31—P2—C25—C26	−63.51 (17)
C13—P1—C7—C8	78.37 (17)	C19—P2—C25—C26	179.81 (15)
N1—P1—C7—C12	20.69 (17)	C30—C25—C26—C27	1.5 (3)
C1—P1—C7—C12	148.16 (15)	P2—C25—C26—C27	−177.65 (15)
C13—P1—C7—C12	−96.84 (16)	C25—C26—C27—C28	−0.1 (3)
C12—C7—C8—C9	0.9 (3)	C26—C27—C28—C29	−1.3 (3)
P1—C7—C8—C9	−174.29 (15)	C27—C28—C29—C30	1.4 (3)
C7—C8—C9—C10	0.7 (3)	C28—C29—C30—C25	0.0 (3)
C8—C9—C10—C11	−2.1 (3)	C26—C25—C30—C29	−1.5 (3)
C9—C10—C11—C12	1.9 (3)	P2—C25—C30—C29	177.68 (14)
C10—C11—C12—C7	−0.3 (3)	N1—P2—C31—C32	44.97 (17)
C8—C7—C12—C11	−1.1 (3)	C19—P2—C31—C32	−74.47 (16)
P1—C7—C12—C11	174.25 (15)	C25—P2—C31—C32	170.70 (14)
N1—P1—C13—C18	2.28 (17)	N1—P2—C31—C36	−133.64 (16)
C1—P1—C13—C18	−123.61 (15)	C19—P2—C31—C36	106.93 (16)
C7—P1—C13—C18	120.73 (15)	C25—P2—C31—C36	−7.91 (18)
N1—P1—C13—C14	−178.09 (14)	C36—C31—C32—C33	0.1 (3)
C1—P1—C13—C14	56.02 (17)	P2—C31—C32—C33	−178.58 (15)
C7—P1—C13—C14	−59.64 (17)	C31—C32—C33—C34	−0.5 (3)
C18—C13—C14—C15	0.2 (3)	C32—C33—C34—C35	0.2 (3)
P1—C13—C14—C15	−179.39 (14)	C33—C34—C35—C36	0.4 (3)
C13—C14—C15—C16	−0.2 (3)	C34—C35—C36—C31	−0.8 (3)
C14—C15—C16—C17	−0.4 (3)	C32—C31—C36—C35	0.6 (3)
C15—C16—C17—C18	0.9 (3)	P2—C31—C36—C35	179.15 (15)
